# Extended urbanisation and the spatialities of infectious disease: Demographic change, infrastructure and governance

**DOI:** 10.1177/0042098020910873

**Published:** 2020-03-31

**Authors:** Creighton Connolly, Roger Keil, S. Harris Ali

**Affiliations:** University of Lincoln, UK; York University, Canada; York University, Canada

**Keywords:** demographic change, extended urbanisation, governance, infectious disease, infrastructure, urban political ecology

## Abstract

This paper argues that contemporary processes of extended urbanisation, which include suburbanisation, post-suburbanisation and peri-urbanisation, may result in increased vulnerability to infectious disease spread. Through a review of existing literature at the nexus of urbanisation and infectious disease, we consider how this (potential) increased vulnerability to infectious diseases in peri- or suburban areas is in fact dialectically related to socio-material transformations on the metropolitan edge. In particular, we highlight three key factors influencing the spread of infectious disease that have been identified in the literature: demographic change, infrastructure and governance. These have been chosen given both the prominence of these themes and their role in shaping the spread of disease on the urban edge. Further, we suggest how a landscape political ecology framework can be useful for examining the role of socio-ecological transformations in generating increased risk of infectious disease in peri- and suburban areas. To illustrate our arguments we will draw upon examples from various re-emerging infectious disease events and outbreaks around the world to reveal how extended urbanisation in the broadest sense has amplified the conditions necessary for the spread of infectious diseases. We thus call for future research on the spatialities of health and disease to pay attention to how variegated patterns of extended urbanisation may influence possible outbreaks and the mechanisms through which such risks can be alleviated.

## 摘要

本文认为，当代的扩展城市化进程，包括郊区化、后郊区化和周边城市化，可能会导致传染病传播方面脆弱性的增加。通过回顾现有的关于城市化和传染病之间关系的文献，我们研究这种（潜在的 ）周边或郊区传染病脆弱性的增加与大都市边缘的社会物质转变之间是如何辩证相关的。特别是，我们强调了文献中确定的影响传染病传播的三个关键因素：人口变化、基础设施和治理。鉴于这些主题的突出性及其在城市边缘疾病传播中的作用，我们选择了这些主题。此外，我们提出了景观政治生态框架在研究社会生态转变对周边和郊区传染病风险增高的影响方面的作用。为了说明我们的论点，我们将使用世界各地各种重新出现的传染病事件和爆发的例子，揭示了最广以上的城市化扩展如何扩大了传染病传播的必要条件。因此，我们呼吁，在未来对健康和疾病的空间关系的研究中，应关注各种不同的扩展城市化模式对疾病爆发风险的影响，以及减轻这种风险的机制。

## Introduction

To date, the literatures on urbanisation and globalisation have focused primarily on economic and demographic flows to, from and through cities and their regions (Brenner, 2014; Ren and Keil, 2017). More recently, there has been a growing academic and policy interest in connecting challenges of a majority urbanised world to questions of health and disease, including Ali and Keil’s (2008) edited collection, *Networked Disease: Emerging Infections in the Global City*, as a ground-breaking publication (see also Elsey et al., 2019; Moore et al., 2003; Wu et al., 2017). Meike Wolf’s (2016) research on urban epidemiology also takes forward the debate surrounding the role of changing geographies on urban health and disease. Furthermore, a recent article by Bollyky (2019) has more explicitly noted that the future of global health is urban health. Recognising these emerging conversations, we are specifically interested in this paper in new ways in which infectious disease is bound up with processes of *extended* urbanisation, paying particular attention to the socio-ecological flows and disruptions leading to an increased incidence of infectious disease in peri- or suburban areas.

This paper, then, will focus on the impact that more extensive forms of urbanisation worldwide have on increasing susceptibility to infectious disease, especially emerging infectious disease (EID), that is, ‘an infectious disease whose incidence is increasing following its first introduction into a new host population’ (Quammen, 2012: 43), and zoonosis, which is ‘an animal infection transmissible to humans’ (Quammen, 2012: 14). Notably, ecological pressures coupled with social and spatial change have led to new forms of disease spread that have likewise contributed to the rise of EID epidemics. These include changes in water-borne EID spread, as was the case with *E. coli* 0157:H7 (Ali, 2004); changes in food-borne EID transmission as brought on by changes in global consumption patterns (Hoffman, 2014); as well as changes in the distribution of vector-borne diseases such as malaria, wherein the distribution of mosquitos has been affected by global climate change (Brisbois and Ali, 2010; Epstein, 1998; Nading, 2014).

In examining these relationships between extended urbanisation and infectious disease we suggest how a landscape political ecology (LPE) framework can be useful for integrating the key themes of spatiality, socio-natural metabolism and power relations central to undertaking such an analysis. As we discuss, LPE approaches are crucial to understanding the metabolic processes and socio-environmental implications bound up with extended forms of urbanisation. The concept of metabolism refers to the combination of social and natural processes to form socio-natural landscapes (Swyngedouw and Heynen, 2003). We posit that, while rapid and intensive forms of urbanisation (densification) are seen as enabling factors for the spread of infectious disease (Munster et al., 2018), it is important to study extended urbanisation because patterns of urban sprawl and expansion are more likely to lead to infectious disease outbreaks, as opposed to cities, which are generally assumed to reduce the incidence of infectious disease for inhabitants (see Wood et al., 2017). This is, in part, because urban expansion might expose sub- and ex-urban areas to higher levels of biodiversity (and disease sources) than are found in central urban areas (Kaup, 2018). Cities also have better health facilities and resources which can enable faster response times and enhance containment of disease outbreaks.

In other words, the new and evolving global peripheries have been particularly susceptible to diseases that jump the animal-to-human species boundary (zoonosis); have seen the introduction of new disease vectors; and have seen dynamic changes to urban and spatial morphology and transformations over time (Brisbois and Ali, 2010). In demonstrating this argument, we highlight three key factors influencing the distribution of infectious disease burden that have been identified in the literature on this topic within various strands of urban studies. These interrelated dimensions are mobility and demographic change, infrastructure and governance. There are certainly more factors that could be identified (e.g. deforestation and climate change) but these three themes have been most prominent in the literature on urbanisation and infectious disease, and also relate closely to processes of extended urbanisation.^1^ We have kept these themes intentionally broad to capture as much of the disparate work that exists on this topic as possible. Moreover, we consider how these factors influence different phases of infectious disease management, from disease prevention to mitigation and control of outbreaks, and possible responses.

In what follows we will first establish the context for this paper, outlining why the relationship between extended urbanisation and infectious disease is important to study, and how this differs from existing work on the relationship between urbanisation and disease. Subsequently, we introduce the conceptual framework of landscape political ecology, indicating how this relates to (but differs from) urban political ecology and how this is a useful lens through which to understand the emergence of infectious disease in peri-urban areas. We then survey the three themes of mobility and demographic change, infrastructure and governance, in turn, discussing their importance for addressing emerging urban challenges related to extended urbanisation. Finally, we reflect on how the expansion of the city can influence the spread of disease and how this can be addressed in future research.

## Extended urbanisation and emerging infectious disease

Contemporary patterns of extended urbanisation fundamentally shift the vulnerability of cities to infectious diseases in ways that differ from those that have historically been associated with urbanisation. Such processes of urban expansion are linked to the ubiquitous reordering of the global urban periphery through complex processes of displacement of central populations to the margins and the creation of new functional centralities (jobs, infrastructures, densities) away from the traditional core. As indicated, we use the term extended urbanisation as a summary concept for these developments. The processes captured under this term, originally informed by the urban theory of Henri Lefebvre (2003), predict what he called ‘the complete urbanisation’ of society. This phenomenon is partially caused by the rapid growth of the human population and the expanding geographical reach of capitalist accumulation over the past century, which have brought about an ‘urban revolution’ and the creation of an ‘urban society’ at the planetary scale (see Keil, 2018a). Relatedly, various scholars have argued that we are now witnessing a process of planetary urbanisation, which is premised upon expanding infrastructural networks and human settlements (Brenner, 2014).

In this broader context, we are specifically interested in what Lefebvre (2003) calls the spread of ‘the urban tissue’ across the planet, which refers to the fluid relationships between urban and rural environments. Forms of extended urbanisation – such as suburbanisation – are an empirically recognisable process in this context. In many parts of the world, particularly in the Global South, peri-urbanisation is the preferred term for extended urbanisation (de Vidovich, 2019). Some scholars have called the current phase of urban extension ‘post-suburbanisation’, which leads to an increasing complexity of structural form and daily life in the periphery of cities (Charmes and Keil, 2015; Wu and Phelps, 2011). In this context, ‘peripheral’ can also refer to both the self-built structures and the informal communities that characterise much of today’s urbanisation without being necessarily spatially on the margins (e.g. refugee settlements, mining camps and indigenous reserves near urban centres) (Caldeira, 2017; Güney et al., 2019). Finally, extended urbanisation refers to new and existing urbanisation and urban settlement in the periphery of cities *and* relations that condition these spaces but also reach beyond them (e.g. mines, factories and infrastructures) (Keil, 2018a).

Such patterns of urbanisation – including connected processes of globalisation and neoliberalisation – can increase the qualitative conditions and the statistical odds that microbes are being spread, which has resulted in a tripling of the total number of disease outbreaks per decade since the 1980s (Ali and Keil, 2007; Haggett, 1994). As Wald (2008: 14) has put it, cities have been known by public health officials as ‘promiscuous’ social spaces, with people ‘literally and figuratively bumping up against each other in smaller spaces and larger numbers than ever before’. Additionally, the most significant global disease outbreaks in recent years have originated in China and Africa, which are also amongst the most rapidly urbanising regions (Alirol et al., 2010). Both SARS and Ebola originated in urbanising hinterlands before travelling to and spreading in and between major cities such as Hong Kong and Toronto or Freetown and Monrovia, respectively (Keil and Ali, 2007). As such, how and why the proliferation of suburban or peri-urban areas is conducive to disease spread is an important question to explore.

While other approaches situate communicable disease as a function of social interactions, we focus on changing spatial factors that drive changing patterns of disease. This can be cast as part of a general concern with the spread of risk as processes of peri-urbanisation and suburbanisation are arguably the defining forms in which global urban society is taking shape in the 21st century. In this context, Bloch et al. (2013: 96) observed that ‘current urban growth patterns appear to have significantly amplified the exposure of urban populations to hazard risks, markedly but not exclusively those broadly characterised as the urban poor’. Therefore, identifying areas where the convergence of risk factors is occurring with greatest intensity, and at the largest scales, is a logical first step in the development of a mitigation strategy.

### Chronic and (emerging) infectious disease

Before we continue, we need to acknowledge that much of the debate about urbanisation or, more precisely, extended urbanisation and health/disease is occupied by a burgeoning interest in chronic diseases associated with a lifestyle that is ascribed to suburbanisation, auto-mobility and related technologies. At the top of the list of these concerns are usually obesity (especially amongst young people), diabetes and heart disease (Hamblin, 2014). Importantly, attention has now also shifted to mental health related to suburban life, for example in emerging work on people living with dementia in suburban environments (Biglieri, 2018). Much of the literature on chronic disease has, as the tacit starting point, the notion of ‘epidemiological transition’. That is, the argument that in Western, industrialised societies, more individuals are living to older ages, which consequently leads to increased incidence and prevalence of chronic diseases associated with (sedentary) ‘lifestyle’ and ‘ageing’, as opposed to infectious diseases. Bloch and co-authors (2013: 96) of course warn: ‘A closer examination of urban risk shows, in fact, that “sprawl” is not the problem, but rather the lack of adequate land use planning policies and infrastructure provision in rapidly growing and expanding settlements.’

One of the key points of emphasis in approaches based on this premise is a focus on the physical environment found within such contexts. Much of the health research on the built environment reveals that the health of those in urban areas tends to be worse than that of those residing in less urbanised areas – a disparity referred to as the urban health penalty (Freudenberg et al., 2005). However, such findings are not wholly conclusive, as research has also pointed to certain features of urban life that benefit the health of urban dwellers, including the availability of social support and better access to health and social services (Bollyky, 2019). Current health research does conclusively demonstrate, however, that one aspect of the built environment is detrimental to good health, namely, living in sprawling suburban neighbourhoods (Freudenberg et al., 2005; Frumkin et al., 2004). What has made this question more complicated is exactly the tendency, invoked in our usage of extended urbanisation and post-suburbanisation, of blurring the classical lines of distinction of city and suburb, town and country. It appears more important for the health of communities and individuals where in the world and, indeed, where in the urban region they are located and how those particular areas are changing in relation to their natural and social environments (Wilson et al., 2008).

As alluded to above, the tendency to date not to have focused concerted attention on infectious disease may have to do with the ‘epidemiological transition’ model. According to this perspective, Western societies have undergone a health transition whereupon infectious diseases were no longer to be considered as major causes of mortality and morbidity (Omran, 1971). Thus, for example, it was in this light that in 1967 the US Surgeon General publicly declared that it was ‘time to close the books on infectious diseases’ and to shift all national attention to chronic diseases such as cardiovascular disease (Garrett, 1994: 33). Recent developments, however, appear to indicate that this may be premature as we now appear to face an onslaught of what are referred to as new and (re)emerging diseases (Garrett, 1994; Haggett, 1994; Mayer, 2000; Morse, 1996).

Emerging diseases are those which have become more prevalent during the last quarter century, while ‘new diseases’ refers not only to newly appearing ones but also to those that are spreading to new geographical areas (Mayer, 2000). Some examples of these include: yellow fever, the Marburg virus, Legionnaires’ disease, the Ebola virus, Lyme disease, hepatitis C, HIV/AIDS, Hantavirus pulmonary syndrome, West Nile virus and Severe Acute Respiratory Syndrome (Drexler, 2002; Garrett, 1994; Heymann and Rodier, 1997). On the other hand, re-emerging diseases, or those thought to have been eradicated through aggressive antibiotic vaccination campaigns, have also begun to reappear with greater frequency in the population in recent years (for example, tuberculosis).

## Landscape political ecologies of health and disease

Spatialised political ecology of health approaches – paying specific heed to interactions between urban, suburban and rural landscapes – are important for their focus on the interaction between political interests, social institutions and the human–non-human environment, which can bring about a greater systemic understanding of health and disease (see Connolly et al., 2017; Jackson and Neely, 2015; King, 2010). Given the interdisciplinary nature of health studies, political ecology is an ideal framework that allows for the use of mixed research methods and incorporates a range of conceptual approaches (King, 2010; Robbins, 2012). This is because of its deep concern for human/environment relations, and for its systematic study of the unequal distribution of socio-environmental harms and risks. More specifically, Connolly (2017) suggests that a landscape political ecology (LPE) perspective can be a useful approach for examining the political ecologies of disease. This is because both political ecology and health geographies draw on ideas of place and landscape and utilise an understanding of place as a socially (re)constructed phenomenon (see King, 2010).

The concept of landscape is useful for studying processes of extended urbanisation, given the hybrid nature of the term, which allows for blurring distinctions between the urban and rural. This is one way in which sub/urban political ecologies have moved beyond critiques of ‘methodological cityism’, by exploring socio-ecological processes on the urban periphery (c.f. Angelo and Wachsmuth, 2015; Connolly, 2019). The landscape lens is also important to understand how spatial factors and the physical ordering of the urban environment can directly influence the incidence of disease outbreaks and possible responses to them (Lambin et al., 2010). Moreover, Kearns and Moon (2002: 611) have argued that landscape serves as a metaphor for ‘the complex layerings of history, social structure and built environment that converge in particular places’. This can be seen in the infrastructural (dis)connections and changing nature–society interactions that are associated with urban expansion. As such, we posit that particular landscapes themselves can be structured in such a way that they influence the likelihood of disease transmission.

Some scholars working on the political ecology of health and disease have used landscape as an analytical lens to consider how various health discourses can become materialised in particular places (Mulligan et al., 2012; Parizeau, 2015). For example, Wald (2008: 2) has described how ‘the circulation of microbes materialises the transmission of ideas’ regarding theories about how diseases spread, and attitudes toward social change. In this way, disease is not only determined through biophysical factors but also constructed out of a particular set of social and spatial relations which are mediated through the landscape. As we will discuss later in the paper, processes of extended urbanisation can increase risk from infectious diseases – which are themselves rapidly evolving – as the nature and mode of transmission are often neither well understood by science nor properly regulated by government, particularly in informal peri-urban settlements common in the developing world. Lambin et al. (2010) have examined specifically how landscape attributes and land use change can have a significant impact on re-emerging infectious diseases and/or zoonoses.

Others have used a landscape lens to examine the interconnections between social and environmental systems (Fairhead and Leach, 1996; Walker and Fortmann, 2003). In this regard, Wald (2008: 2) has observed how interactions between microbes, bodies and spaces have the tendency to blend together as they ‘animate the landscape and motivate the plot of the outbreak narrative’. Such analyses draw upon a wide body of literature in science and technology studies (STS) and are influenced by assemblage theory exploring the agency of non-humans in shaping urban environments and the regulation of public space (see Braun, 2008; Jackson and Neely, 2015; Rose, 2007). Urban political ecology has also mobilised insights from STS to analyse the role of non-humans in shaping human health (see Jackson and Neely, 2015). This has been achieved through the use of analytical and heuristic concepts such as Haraway’s ‘cyborgs’ (Haraway, 1991) and Latour’s ‘quasi-objects’ (Latour, 1993) – terms that are now commonplace in the literature on urban political ecology and in the social sciences more broadly.

Braun (2008), for example, has argued that infectious diseases emerge from human–non-human relationships, circulation and exchange at a variety of scales from the molecular to the global. This has caused various non-human animals including rodents to enter human settlements, which is partly to blame for the first Ebola case in Guinea. Processes of extended urbanisation have also facilitated the expansion of human settlements into former rainforest areas, exposing humans to new possible sources of disease (see Yong, 2018). Deforestation and human encroachment on wildlife habitats have increased interactions between wildlife, human beings and livestock, thus heightening the potential for pathogens to cross the species barrier (Coker et al., 2011). As Yong (2018: n.p.) has explained, such patterns have now become the general context for the spread of infectious disease resulting from zoonotic infection, noting that: ‘wherever people push into wildlife-rich habitats, the potential for such spillover is high’ (Yong, 2018). Such processes have been facilitated by the greater and more rapid movement of people, exposing human populations to a host of microbes, insects and other non-human organisms which were previously largely undisturbed by urbanisation.

Maria Kaika’s (2005) city of flows analogy, born out of the urban political ecology (UPE) literature, is also useful for conceptualising the relationship between extended forms of urbanisation and disease. UPE examines the multitude of socio-natural flows into and out of the city, often referred to as ‘urban metabolism’, including biophysical, technical, social and economic exchanges (Gandy, 2004; Loftus, 2006; Swyngedouw, 2006). Kaika and colleagues have also recently proposed a distinctive suburban political ecology lens which has considerable overlap with the perspective put forward here in our combined use of landscape and urban political ecologies (Tzaninis et al., 2020). As Coker et al. (2011: 599) have elaborated, cities are home to ‘dynamic systems in which biological, social, ecological and technological processes interconnect in ways that enable microbes to exploit new ecological niches’. Moreover, ‘the particular sociopolitical contexts and spatial configuration of urban regions have strong implications for how these various non-human natures are urbanised’ (Connolly, 2019). For these reasons, landscape political ecology becomes an extremely useful tool for understanding the political, social, economic and cultural relationships between urban environments and public health.

## Extended urbanisation and infectious disease: Three dimensions

Meike Wolf (2016: 975) has proposed a number of ‘future challenges’ of research into the ‘messy materialities’ of (extended) urbanisation and (emerging) infectious disease research. In summarising her review of recent developments in the field, Wolf argues that ‘a reconsideration of analytical categories of space, time, climate or nature – which are of equal importance to both the social sciences and public health – goes hand in hand with accounts that ramify different sites and aim to capture new paths of connection and association’ (Wolf, 2016: 976). This can be more useful than making overarching processes such as globalisation or urbanisation synonymous with increases in mobility. With this in mind, we have isolated three dimensions of possible research on suburbanisation and infectious disease: dynamics of population change, infrastructure and governance. As we will demonstrate, these concepts are well suited to a landscape political ecology approach and are crucial for identifying spatial patterns and political-economic arrangements that influence the spread of infectious disease in the ongoing, and accelerating, process of extended urbanisation.

### Dynamics of population change

Dying alone in your hut isn’t an outbreak. (Khan and Patrick, 2016: 70)

This terse statement by former director of the US Centers for Disease Control’s Office of Public Health Preparedness and Response points the finger at an obvious truth: pandemic disease relies on population growth. Population growth in cities – driven primarily by rural–urban migration – is a major factor influencing the spread of disease (Coker et al., 2011). This is seen most clearly in rapidly urbanising regions such as Africa and Asia, which have experienced recent outbreaks of Ebola and SARS, respectively. Projections by urban scholars hold that sub-Saharan Africa’s urbanisation rates are higher than anywhere else in the world as the urban population in the region ‘is expected to quadruple, from 295 million to 1.15 billion’ (Angel et al., 2017: 169). Twelve million people now live in Kinshasa, capital of the DRC, which is three times the combined population of the cities affected by the 2014 outbreak in West Africa (Yong, 2018). Equally, regional towns in the DRC, where some of the recent Ebola cases have been recorded, have also been expanding, some under the influence of conflict and war. While the ecological consequences of this expansion are beginning to be better understood, we are only starting to shed light on the impact of dramatic and massive sub/urbanisation on health and disease.

But, studies at the intersection of urbanisation and infectious disease have shown that it is not only population growth that leads to infectious disease spread but also density. Jakarta, to take another example, is projected to become the largest city in the world in coming decades, with much of the population made up of rural–urban immigrants. Numerous researchers have thus noted that population density – which is highest in cities – strongly influences the likelihood of a disease outbreak (Ali and Keil, 2007; Alirol et al., 2010; Coker et al., 2011). For instance, Wilkinson and Leach (2015) have noted that the dense urban areas and slums in Monrovia and Freetown, Sierra Leone, have been prime sites where Ebola has thrived. While suburban areas are popularly understood as low density areas, such processes of extended urbanisation in developing regions often consist of densely populated ‘new towns’ of high-rise flats or peri-urban informal settlements with high densities (see Mabin et al., 2013). Such cases indicate the importance of a landscape political ecology lens in examining urbanisation and infectious disease, as it is often the lack of physical infrastructure coupled with political-economic factors resulting in high density in such places that provide perfect scenarios for the spread of microbes.

Research on urbanisation is also beginning to consider how mobility patterns between urban, peri-urban and rural areas influence infectious disease spread (see Herrick, 2014; Wolf, 2016). It should be noted that the first urban Ebola outbreaks happened in West Africa after almost four decades of rural outbreaks throughout the rest of Africa (WHO, 2015). Why, then, was there a change from rural to urban outbreaks after this time and in this particular region? One factor is the high degree of population movement on the continent, which is seven times higher than anywhere else in the world (WHO, 2015). This migration is driven by a myriad of social and political-economic factors that force people to travel daily in search of food or work; extended families with relatives living in different countries; the traditional practices of returning to a native village to die and be buried near ancestors; as well as travel to traditional healers who have the trust of community members (WHO, 2015). There are also the effects of civil war that have forced some family members to flee their home villages to other, usually more urban areas, for relocation and resettlement.

Disease transmission in large urban populations can also be affected by heterogeneity in health of urban dwellers, increased rates of contact, and mobility of people (Alirol et al., 2010). For instance, Alirol et al. (2010) and Tong et al. (2015) have shown that rural to urban population movements can substantially increase risk of transmission amongst newcomers who may not have previous exposure (immunity). It is also difficult to control migration between cities in many African countries, as Sierra Leone, Liberia and Guinea each have 5000 border crossing points (Wilkinson and Leach, 2015). Thus, the monitoring of rural–urban and inter-urban migration will be crucial in order to stop the spread of disease in future outbreaks. Tong et al. (2015: 11,029) further add that rural–urban migrants tend to be poorer and less educated than the permanent population in urban areas, live in lower quality housing with inadequate sanitation, have limited access to health services (see also Hynie, 2018). These migrants tend to settle in (often informal) places along the metropolitan edge. This can be problematic, as Wu et al. (2017: 21) have found that in many Chinese cities, public health management has not kept pace with demographic changes in rapidly urbanising areas.

As Wolf (2016: 965) has noted, infectious diseases are thus less of a ‘natural’ disaster, but emerge alongside social and spatial inequalities in housing, health education or financial resources (see Kotsila, 2017). Such processes are particularly well suited to an urban political ecology framework which is not only useful for examining the ‘explosion’ of urban societies, but also the uneven and socially unjust power relations which amplify health inequalities in particular places, and underlines the issue of governance that we will deal with later (see Houston and Ruming, 2014; Parizeau, 2015). Understanding the root causes of disease emergence in urban areas will thus be essential to preventing additional rural to urban spread and to containing outbreaks within urban centres (Fallah et al., 2018: 280; Richards et al., 2015).

### Infrastructure

Viruses have no locomotion yet many of them have traveled around the world. (Stephen S Morse, in Quammen, 2012: 24)

In many ways, the geographic spread, growing sophistication and colonising propensities of transportation networks are the hallmark of extended urbanisation in general (Keil, 2018a). Specifically, peri-urban (transport) infrastructures are tremendously important for the functioning of the entire urban region (Filion and Pulver, 2019). This is due to the location of prime network spaces such as airports and recreational spaces, in addition to the noxious or toxic industrial infrastructures including waste and water treatment facilities and incinerators, which are often in peri- or suburban areas (Keil, 2018b: 132). Diseases can spread rapidly between cities through infrastructures of globalisation such as global air travel networks. While this has been well documented before, it is relevant here because airports and other nodes of economic logistics and activity are often located in suburban municipalities, thus raising potentially complex governance and jurisdictional issues with regards to who has responsibility to control disease outbreaks in large urban regions (Addie, 2014; Ali and Keil, 2010; McNeill, 2011).

Ex-urban infrastructures have thus become the lynchpin of urban mobility and circulation and of socio-natural metabolisms (see Filion and Pulver, 2019; Lin, 2019; Monstadt, 2009). As Lin (2019: 76) writes, ‘infrastructures often figure as networked landscapes, constituting “spatial products” that script structural relations between places at the planetary scale’. For this reason, such infrastructures can facilitate the transmission of infectious diseases and make urban populations more vulnerable (Keil and Ali, 2007: 848). Indeed, the spread of disease is enabled by the same infrastructures that carry people, resources and goods. For instance, Munster et al. (2018) have argued that road construction for logging, mining and hydroelectric activities ‘continues to open access to remote locations’, making road development between major or minor urban centres a key factor in the spread of infectious disease.

Transportation infrastructure is thus a primary form of ex-urban infrastructure which can lead to the spread of disease, particularly in outbreak situations (Keil and Ali, 2011: 131). During the recent outbreaks of Ebola in Central and Western Africa the increasing quality of transportation infrastructure, connecting African cities with each other and the world, have been seen as a decisive factor (McNeil, 2011). While Ebola is nothing new to the affected regions, previous cases have been contained by poor transport infrastructure, making travel between cities very challenging and time consuming. As Yong (2018) recently observed, the paving of the road between Kikwit and Kinshasa in the DRC decreased travel time from more than a week to just eight hours. Affected patients would thus leave Kikwit for Kinshasa seeking treatment, which could infect more people in Kinshasa. Such connections illustrate the central role of landscape in connecting infrastructures and local environments, and the ‘enviro-technical assemblages’ that can influence socio-ecological processes and the spread of infectious disease (see Ali and Keil, 2010; Houston and Ruming, 2014; Keil and Young, 2009).

We also need to take into account the *disconnections* that become apparent as rapid demographic and peri-urban growth is not accompanied by appropriate development of social and technical infrastructures. The rapid pace of urban expansion has meant that many emerging and existing ex-urban landscapes contain ‘infrastructure deserts’, especially in the Global South, as infrastructure development has not been able to keep up with the spread of population (Keil, 2018b: 139). For example, Wilkinson and Leach (2015) have noted that the ‘precariously expanded urban areas’ in West Africa have become populated by unemployed young people and lack basic municipal planning and services including access to fresh water, or have poor sanitation which would increase the potential threat of water-borne disease and low health indicators. Coker et al. (2011: 603) similarly found that population growth and urbanisation in South-east Asia have meant the number of people using unimproved sanitation and drinking water systems in urban areas has risen by 20 million between 1990 and 2006. Finally, as Kotsila (2017: 99) found, there is a ‘considerable number’ of people in Can Tho City who lack access to piped water but are statistical minorities and as such do not receive as much attention as those in rural areas.^2^

Therefore, Filion and Keil (2017) have argued that it is important to study suburbs in particular because of their rapid growth rate, which is often coupled with an insufficient infrastructure development response. This echoes Mulvihill and Ali’s (2007: 356) observation underscoring the vulnerability of ex-urban places to an intensifying ‘urban shadow’ along the urban periphery. This is especially true in the Global South, particularly in informal settlements whose needs are overlooked by governments, combined with lower income of residents. For example, Zhang et al. (2008) have highlighted the paucity of studies in developing countries which study the relationship between urbanisation and disease. However, ex-urban areas in developed regions are also rapidly growing and can likewise be vulnerable if there is little knowledge about how to control a particular disease. This was evidenced in the case of an American healthcare worker who contracted Ebola in Dallas, Texas, through treating an infected patient who had recently returned from West Africa (Courage, 2014).

### Governance

The crucial issues of governance and political-economic factors in relation to infectious disease have been a topic of scientific analysis since the middle of the 19th century, when Rudolf Virschow and John Snow demonstrated the connection between socio-economic context, natural resource management and outbreaks of various epidemics (Connolly et al., 2017: 3). Subsequently, health and medical geographers have examined the political-economic factors shaping the spatial distribution of disease in order to achieve a more systemic understanding of health (see Haggett, 1994; Kearns, 1993; Mayer, 1996). In particular, scholars such as Mayer (2000) and Ali (2004) have demonstrated how certain sociopolitical conditions associated with a physical setting can act as structural causes that play a central role in the number and intensity of disease outbreaks in a given area. In terms of extended urbanisation, this means that disease response mechanisms and other forms of governance may not be as well established in peri-urban areas, resulting in increased vulnerability to disease outbreaks.

One concept through which scholars have addressed the relationship between governance and disease is that of biopolitics, which refers to the ways in which health and disease have historically been closely associated with the modern (nation) state and its politics of governing (Braun, 2008; Collard, 2012; Rose, 2007). We cannot discuss this history in detail here. However, it has a bearing on our discussion directly through its link with settlement, urbanisation and density. In particular, it describes how the state controls populations for various purposes, including ostensibly for the purpose of disease management. Examples include public health, town planning and administration, which seek to ‘improve’ the national population by eliminating risks to its future wellbeing (Braun, 2008). As Collard (2012) notes, biopolitical approaches examine how safe space is made, maintained and unmade, and how non-humans (e.g. animals, bacteria, zoonoses) matter to the material and semiotic construction of ‘safety’ and space. As discussed above, urban political ecology approaches also discuss the ways in which governance decisions result in unequal and spatialised patterns of disease whereby particular spaces and population groups face a disproportionate burden of disease for various reasons (Rose, 2007; Sarasin, 2008).

In this context, political ecology is a useful framework for considering issues of governance given that political economy and power are central to its analysis of the relationships between humans and their environment (Kaup, 2018; King, 2010; Turshen, 1984). For instance, King (2010: 42) has argued that political ecology of health frameworks can illustrate how key actors and institutions and human–non-human relationships can influence the transmission of disease and ability of institutions to provide effective treatment. It can also help to understand how various power relationships and government policies at a variety of scales can reinforce social inequalities that influence vulnerability to disease. Kaup (2018), for instance, has drawn attention to neoliberalisation and privatisation or rolling back of government services as a factor influencing disease outbreaks, particularly in ex-urban areas. As he notes, this results in a decreased state ability to respond to outbreaks when they occur and to create conditions in which outbreaks are less likely.

Future studies on extended urbanisation and infectious disease could therefore examine how government policies might seek to regulate patterns of sub- and ex-urbanisation in the interests of ‘healthy cities’. One area of focus here should be on the changing composition of ex-urban populations and communities including the phenomenon of the ‘suburbanisation of poverty’, which brings new health concerns to areas that had traditionally been seen as privileged and well served by public health agencies and private providers of healthcare (Kneebone and Garr, 2010). Highlighting spatial inequalities in healthcare provision and response in urban areas is a topic which is well suited to urban political ecology frameworks, given the field’s focus on environmental injustice.

Relatedly, the notion of ‘political pathology’ has also been relevant in the governance of infectious disease. David Fidler (2004), in particular, has put forward the notion – with respect to the SARS epidemic in 2003 – that this ‘first severe infectious disease to emerge in the 21st century’ was also the harbinger of a changing global landscape of health governance. Fidler argued that ‘SARS is the first post Westphalian pathogen because its nonrecognition of borders transpired in a public health governance environment radically different from what previous border hopping bugs encountered’ (Fidler, 2004). Importantly, governance now had to recognise that the classical nation-state-centric approach to global health had to adapt to changing realities in a world that became both more transnational and more localised. The debate on global health security has since been constantly in the foreground of governance on a rapidly changing planet especially after recent Ebola outbreaks in West Africa and the DRC (Halabi et al., 2017).

As Priscilla Wald (2008: 17) has observed, drawing upon Rosen’s (2015 [1958]) earlier work on the history of public health in Europe, epidemics ‘dramatise’ the need for regulation with ‘terrifying urgency’. They further put in place the ‘administrative machinery for disease prevention’ and protection of public health (Rosen, 2015: 47). As Keil and Ali (2011) found, it was typically conventional containment strategies, such as isolation and home quarantine, that proved most successful for controlling the spread of SARS in affected cities. As they note, this is based on the view of the bounded city with fixed, territorialised and restricted access, which contrasts with the unbounded and ‘topological’ character of contemporary urbanisation processes. The coronavirus (COVID-19) epidemic that spread just as we were completing this paper sparked the ‘largest quarantine in human history’, resulting in the isolation of entire cities and regions (Gollom, 2020). Wuhan’s urban periphery also became the setting for a ‘pop-up’ construction of a 1000-bed hospital facility to deal with affected patients. As Wuhan is locally known as the ‘thoroughfare of China’ (Huifeng, 2020), such spatial factors account for the need for a landscape political ecology approach that can interrogate the relationships between social actors across multiple spatial and temporal scales.

While the governance of disease control and prevention has often taken place at a municipal scale, the increasing porosity between urban, suburban and peri-urban places requires a new approach (see Houston and Ruming, 2014). Cities are thus reconceptualised ‘as unbounded and polyrhythmic spaces, no longer understood in terms of fixed locations in abstract space, but rather in terms of a continuously shifting skein of networks, with their own spatiality and temporality’ (Ali and Keil, 2007: 1217). Thus, the growth of megacities and mega-regions raises the critical question of who has the mandate to control outbreaks in peri-urban areas (see Keil and Ali, 2007). This issue of jurisdictional authority is particularly noteworthy in the context of public health and its connection to the unique type of governance relationships that may exist between urban and ex-urban centres. There is a need for future research in this area, to identify areas for improvement in urban health governance, which will assist in preventing future outbreaks.

## Conclusion

This paper has offered an initial attempt to theorise the relationship between processes of extended urbanisation and infectious disease, while also establishing the basis for a future research agenda in this area. The massive increase of the global urban population over the past few decades has been concentrated primarily in ex-urban areas, which has posed new challenges to the control of infectious disease. This includes processes such as population growth and movement between urban, ex-urban and rural areas, as well as infrastructure provision (e.g. water and sanitation) and land use change. As we have noted, these processes are especially pronounced in (but not limited to) developing regions, which have also been the source of recent major outbreaks such as Ebola and SARS. We have also noted how the governance of infectious disease is challenging, with overlapping institutional roles and responsibilities in urbanising regions, which poses questions as to who should do the work of managing (and preventing) potential outbreaks (see Coker et al., 2011). This is particularly problematic in developing regions, which are often faced with (inter)national political tensions and inequalities that can hinder effective control.

Given the scarcity of research on this topic, there remains a crucial need for both academic research and that which practically informs policy (Coker et al., 2011). In doing this study, we have identified three key areas on which such research efforts should focus, namely: mobility and demographic change, infrastructure and governance. These have been identified based on existing research in these areas, at the intersection of urban studies and infectious disease. These three factors do not constitute an exhaustive list, however, as socio-environmental change – including deforestation and climate change – has been highlighted by authors as a key risk factor which could lead to the emergence of new epidemics and should form the basis of future research (see Brisbois and Ali, 2010; Tong et al., 2015).

We have also illustrated how a landscape political ecology framing which is more attentive to interactions along the urban periphery, can be useful for examining these topics along interdisciplinary lines given the holistic nature of the landscape concept and the diverse methodological approaches comprising political ecology. The attention to socio-ecological metabolisms also allows for understanding how outbreaks of zoonoses and other emerging infectious diseases can be triggered by the expansion of urban settlements in previously forested or agricultural areas. For instance, the aforementioned outbreak of the new coronavirus (COVID-19) first crossed the animal–human divide at a market in Wuhan, one of the largest Chinese cities with 11 million people. As in the SARS pandemic of 2003, the connectivities of accelerated urbanisation, heightened mobilities and more extensive zoonotic risks became immediately apparent (Ali and Keil, 2008).

Such transformations are producing new ecological niches for disease spread, meaning that ex-urban regions are likely to remain a hotspot for EIDs into the foreseeable future. This course of events, continuing as we complete this paper, urges urban researchers to seek new and better explanations for the relationships of extended urbanisation and the spatialities of infectious disease. This will require an interdisciplinary approach including geographers, health scientists, sociologists, while also developing possible solutions to prevent and mitigate future disease outbreaks. As we have argued, landscape political ecology approaches can contribute to this goal by helping to identify the political-economic and biopolitical factors influencing the spread of disease through a range of spatial scales in an age of extended urbanisation.

## Notes

1. As one would expect, this is a highly interdisciplinary field, with contributions not only from social scientists but also epidemiologists.

2. The same is true for waste management in cities, as accumulated waste can be a breeding site for insect-borne vectors (D’Alisa, 2017).
